# Two-Chambered Chitosan Nerve Guides With Increased Bendability Support Recovery of Skilled Forelimb Reaching Similar to Autologous Nerve Grafts in the Rat 10 mm Median Nerve Injury and Repair Model

**DOI:** 10.3389/fncel.2019.00149

**Published:** 2019-05-10

**Authors:** Nina Dietzmeyer, Maria Förthmann, Julia Leonhard, Olaf Helmecke, Christina Brandenberger, Thomas Freier, Kirsten Haastert-Talini

**Affiliations:** ^1^Institute of Neuroanatomy and Cell Biology, Hannover Medical School, Hanover, Germany; ^2^Center for Systems Neuroscience (ZSN) Hannover, Hanover, Germany; ^3^Medovent GmbH, Mainz, Germany; ^4^Institute of Functional and Applied Anatomy, Hannover Medical School, Hanover, Germany

**Keywords:** bendable nerve guides, chitosan, rat median nerve, functional recovery, nerve histomorphometry

## Abstract

Tension-free surgical reconstruction of transected digital nerves in humans is regularly performed using autologous nerve grafts (ANGs) or bioartificial nerve grafts. Nerve grafts with increased bendability are needed to protect regenerating nerves in highly mobile extremity parts. We have recently demonstrated increased bendability and regeneration supporting properties of chitosan nerve guides with a corrugated outer wall (corrCNGs) in the common rat sciatic nerve model (model of low mobility). Here, we further modified the hollow corrCNGs into two-chambered nerve guides by inserting a perforated longitudinal chitosan-film (corrCNG[F]s) and comprehensively monitored functional recovery in the advanced rat median nerve model. In 16 adult female Lewis rats, we bilaterally reconstructed 10 mm median nerve gaps with either ANGs, standard chitosan nerve guides (CNGs), CNGs (CNG[F]s), or corrCNG[F]s (*n* = 8, per group). Over 16 weeks, functional recovery of each forelimb was separately surveyed using the grasping test (reflex-based motor task), the staircase test (skilled forelimb reaching task), and non-invasive electrophysiological recordings from the thenar muscles. Finally, regenerated tissue harvested from the distal part of the nerve grafts was paraffin-embedded and cross-sections were analyzed regarding the number of Neurofilament 200-immunopositive axons and the area of newly formed blood vessels. Nerve tissue harvested distal to the grafts was epon-embedded and semi-thin cross-sections underwent morphometrical analyses (e.g., number of myelinated axons, axon and fiber diameters, and myelin thicknesses). Functional recovery was fastest and most complete in the ANG group (100% recovery rate regarding all parameters), but corrCNG[F]s accelerated the recovery of all functions evaluated in comparison to the other nerve guides investigated. Furthermore, corrCNG[F]s supported recovery of reflex-based grasping (87.5%) and skilled forelimb reaching (100%) to eventually significantly higher rates than the other nerve guides (grasping test: CNGs: 75%, CNG[F]s: 62.5%; staircase test: CNGs: 66.7%, CNG[F]s: 83.3%). Histological and nerve morphometrical evaluations, in accordance to the functional results, demonstrated best outcome in the ANG group and highest myelin thicknesses in the corrCNG[F] group compared to the CNG and CNG[F] groups. We thus clearly demonstrate that corrCNG[F]s represent promising innovative nerve grafts for nerve repair in mobile body parts such as digits.

## Introduction

Traumatic injuries of peripheral nerves represent a frequent reason for morbidity and life-long physical restrictions. Worldwide, about 2.8% of all trauma patients suffer from peripheral nerve injuries (Noble et al., 1998). In many cases, these injuries lead to life-long dependency on aid and substantial reduction of quality of life (Noble et al., 1998). Around the world, more than 1 million people are affected per year (Asplund et al., 2009). Recovery of peripheral nerve injuries often remains incomplete and progresses slowly. Additionally, only less than 50% of the patients regain good to excellent motor functions (Grinsell and Keating, 2014). In clinical routine, peripheral nerve injuries most often affect the upper limbs (Kouyoumdjian, 2006; Deumens et al., 2010) and in 6.4% of all hand injuries, the joint-crossing digital nerves are affected (Renner et al., 2004).

When nerve ends cannot be surgically reconnected by tension-free end-to-end-suture, insertion of autologous nerve grafts (ANGs) still represents the gold standard therapy. However, the usage of ANGs comes along with several downsides such as donor side morbidity, limited availability of donor tissue, and prolonged surgery time followed by increased surgery costs (Hallgren et al., 2013; Faroni et al., 2015).

To circumvent these downsides, we have previously contributed to the development of bioartificial nerve guidance conduits made out of chitosan (Haastert-Talini et al., 2013). In 2015, these nerve guides have been made available for the reconstruction of peripheral nerve gaps in human patients with a maximum distance of 2.6 cm (Reaxon^^®^^ Nerve Guides; Medovent GmbH, Germany). In animal studies, the suitability of those chitosan nerve guides (CNGs) to support peripheral nerve regeneration across limited and critical gap lengths have been proven not only for acute (Haastert-Talini et al., 2013; Gonzalez-Perez et al., 2015; Shapira et al., 2015; Meyer et al., 2016b), but also for delayed (Stenberg et al., 2017) repair of rat sciatic nerves. The rat sciatic nerve injury and repair model is most commonly used to evaluate new developments for peripheral nerve guidance and regeneration support (Angius et al., 2012; Geuna, 2015). When using this model, however, nerve reconstruction is usually performed in an extremity region with minimized mobility, e.g., along the femur bone. In order to address the need for nerve grafts with increased flexibility/bendability in connection with preserved collapse stability for nerve reconstruction in highly mobile regions such as digital joints, we recently modified the original standard CNGs into CNGs with a wavy wall structure at the outside, the so-called corrugated chitosan nerve guide (corrCNG). These corrCNGs have demonstrated similar support as standard hollow CNGs with regard to axonal and functional peripheral nerve regeneration in 15 mm rat sciatic nerve gaps, both after acute and delayed repair (Stößel et al., 2018b). We have previously shown that longitudinal insertion of a perforated chitosan film into hollow CNGs and applying such two-chambered nerve guides (CNG[F]s) for 15 mm rat sciatic nerve gap reconstruction could significantly increase recovery of motor function. However, ANG implantation still resulted in best recovery rates (Meyer et al., 2016a). To further increase the regeneration support given by corrCNGs, we also enhanced them into two-chambered nerve guides by inserting a perforated longitudinal chitosan film (corrCNG[F]) in the current study.

Using the recently advanced rat median nerve injury and repair model (Stößel et al., 2017), we here investigated the regeneration supporting properties of corrCNG[F]s in comparison to ANGs, CNGs, and CNG[F]s after reconstruction of 10 mm nerve gaps. While implantation of ANGs still showed fast and most complete functional recovery, we clearly demonstrate that corrCNG[F]s accelerated and for some skilled-forelimb reaching also significantly increased the recovery of all functions evaluated in comparison to the other bioartificial nerve guides investigated. Results from immunohistological and histomorphometrical evaluation of the distal graft and nerve distal to it underline the superiority of corrCNG[F]s compared to the other artificial nerve guidance channels tested here.

## Materials and Methods

### Experimental Design

In this study, we bilaterally reconstructed 10 mm median nerve gaps of 16 female Lewis rats with either gold standard ANGs (control group; *n* = 8), standard hollow CNGs (*n* = 8), two-chambered chitosan-film enhanced chitosan nerve grafts (CNG[F]s; *n* = 8), or two-chambered corrugated chitosan-film enhanced CNGs (corrCNG[F]s; *n* = 8). This procedure resulted in a 50% reduction of animal numbers for the study, because two reconstruction conditions could be studied in one animal. During 16 weeks of investigation, functional recovery of each paw was separately surveyed using the grasping test (every second week), the staircase test and non-invasive electrophysiological recordings from the thenar muscles (every fourth week). Evaluation was finalized by histomorphometrical analyses of the regenerated nerve tissue within the grafts and distal to it at 16 weeks post-surgery.

### Manufacturing of Classic, Corrugated, and Corrugated Chitosan-Film Enhanced Chitosan Nerve Guides

Medical grade chitosan derived from Pandalus borealis shrimp shells was processed by Chitinor AS (Norway). All types of CNGs were manufactured by Medovent GmbH (Germany) under ISO 13485 regulations using a patented extrusion process with either standard tubular molds or molds with a corrugated design. All nerve guides were prepared in a length of 14 mm, an inner diameter of 1.6 mm, and a final degree of acetylation (DA) of ∼5%. A DA of ∼5% has previously been proven to be most supportive for peripheral nerve regeneration in the rat sciatic nerve model (Haastert-Talini et al., 2013). Chitosan-films for CNG[F]s and for corrCNG[F]s were manually produced as described earlier (Meyer et al., 2016a). Briefly, rectangular pieces (length 10 mm, width: 5 mm) of chitosan-films were perforated (along the midline of the longer axis, diameter of perforation: 0.3 mm, distance in between: 2 mm) and subsequently Z-shape-folded (opposite kinked edges, width of outer edges: 1.7 mm) before they were inserted into the lumen of hollow standard CNGs or corrCNGs leading to 2 mm spaces on each side of the tubes.

Sterilization of all types of CNGs was performed by beta irradiation (11 kGy, 10 MeV) by BGS Beta-Gamma-Service GmbH & Co. KG (Wiehl, Germany) (Stößel et al., 2018a). All CNGs used for median nerve repair were rinsed in 0.9% sodium chloride solution (NaCl 0.9%, B. Braun Melsungen GmbH, Germany) for at least 20 min prior to implantation.

### Animals and Surgical Procedure

This study was carried out in accordance with the principles of the Basel Declaration and recommendations of Directive 2010/63/EU: TierSchG 13.07.2013, BGBl I Nr. 36 12.07.2013, p. 2182 + TierSchVersV. The protocol was approved by the animal care committee of Lower-Saxony, Germany (approval code: 33.12-42502-04-15/1761; approval date: April 10, 2015).

Sixteen young adult female Lewis rats (LEW/OrlRj, mean body weight at the day of surgery: 217.8 ± 1.73 g) were obtained from Janvier Labs SAS [Genest Saint Isle (Le), France] at an age of 13 weeks and housed in groups of four under standardized housing conditions (22.2°C; humidity 55.5%; light/dark cycle 14 h/10 h). Food and water was provided *ad libitum* except during staircase test phases, when animals were fed restrictively with 12 g food per animal and day. As earlier described (Stößel et al., 2017) body weight was controlled every other day (tolerable weight loss up to 15%). A time interval of 72 h was kept between the end of restrictive feeding and eventual anesthesia. Female rats were used because they are generally smaller than male rats (weight difference 100–150 g) and in order to guarantee only minimal size increase during the 16 weeks observation time. Weight increase in Lewis rats follows a flatter curve in female than in male individuals and is already reaching the plateau at an age of 12 weeks in female animals when male rats are still growing. The use of female animals in this study therefore ensured easy handling for the grasping test, when animals were grasped around their trunk with one hand pending to be quickly held only on their tail during the test procedure. The flatter growth curve of female rats further ensured that while already being optimally sized at training onset for the staircase test (not able to turn around or grab pellets with the contralateral paw), tentative weight gain was limited during the observation period. Thus, the staircase test apparatuses did not become too narrow for the still slightly growing young adult animals.

Animals were habituated to the functional testing procedures (grasping test and staircase test) 3 weeks prior to surgery and pre-surgically healthy baseline reference values of all animals were recorded as described in the related sections below.

All surgeries (performed on rats with an age of 19 weeks) and electrodiagnostic recordings were performed under deep anesthesia (intraperitoneal injection of chloral hydrate, 370 mg/kg, Sigma-Aldrich Chemie GmbH, Germany) and aseptic conditions. To minimize the decrease of body temperature animals were placed on a heating pad and rectal body temperature was measured before and after surgery in order to ensure that it did not fall below 36.5°C. After surgery the animals were blanketed with several layers of tissue paper in order to avoid further cooling. Sufficient analgesia during surgery/electrodiagnostic evaluation and the two following days was induced by subcutaneous injection of Butorphanol (0.5 mg/kg, Turbogesic^®^; Pfizer GmbH, Germany).

For nerve transection surgery, a 1 cm incision was performed parallel to the humerus in the axillary region to approach the median nerve. In addition to general anesthesia (see above), drops of bupivacaine (0.25%, Carbostesin^®^; AstraZeneca GmbH, Germany) and lidocaine (2%, Xylocain^®^; AstraZeneca GmbH, Germany) were locally applied on the prospective transection sites of the exposed nerve some minutes before nerve transection in order to ensure sufficient analgesia.

In case of ANG repair, the median nerve was first transected at the distal location (proximal to the point where the median nerve is crossed by the brachial artery), followed by the second transection 10 mm proximal to this point. The nerve piece was then reversed and rotated 180° before suturing it between the two nerve ends. Each end was sutured by two epineural 9–0 stitches. In CNG, CNG[F], and corrCNG[F] groups, the second transection was performed 7 mm proximal to the distal transection with removal of the nerve piece and its further procession for histomorphometrical analyses (healthy control). After application of either nerve guide, both nerve ends were introduced into the nerve graft and sutured with one epineural 9–0 stich generating an overlap of 2 mm at each nerve end and a 10 mm median nerve gap.

Wound closure was performed by using first three to four resorbable sutures on the muscle layers (3–0 Polysorb, UL-215, Syneture, United States) followed by three to four non-resorbable mattress sutures (4–0 Ethilon^TM^ II, EH7791H, Ethicon, United Kingdom) for skin suture.

### Grasping Test – Evaluation of Reflexed-Based Paw-Usage Ability

To evaluate the recovery of finger flexion progressing to restoration of grip force, reflex-based movement (Tupper and Wallace, 1980) was assessed every second week from the fourth week post-surgery onward.

Briefly, the paw-usage ability was video recorded and grip force was measured as described previously (Stößel et al., 2017).

During testing the animals were carefully grasped around their trunk to support them while bringing them close to the test apparatus. For the concrete test procedure the hand around the trunk was removed and the animals were held only by their tail root (1–1.5 cm from the fur) for 5–15 s. Test periods >5 s were only needed when finger flexion was not possible and the experimenter had to take some more time for deciding to end the trial.

For scoring, three categories of grasping behavior were set: Category 1 – no finger flexion while touching the grasping bar; Category 2 – ability to grasp the bar (closing digits around it) but not able to hold it while being slowly withdrawn; and Category 3 – ability to grasp and pull the bar with a detectable force (gross motor skills). Since the scoring was performed in accordance to our previous open access report we kindly refer the reader to it for an illustration (Stößel et al., 2017).

### Staircase Test – Evaluation of Skilled Forelimb Reaching Ability

Prior to surgery (for the recording of healthy baseline reference values) and at pre-defined time points after surgery, the animals were (re-)habituated to the testing procedure and restrictively fed for 7 consecutive days. Therefore, food was restricted to 12 g per animal and day and body weight was controlled every other day. The tolerable weight loss was up to 15%.

On the next 3 days (for pre-surgery values) or on the last 3 days (for post-surgical values), the mean maximum number of pellets retrieved per animal/paw was determined for each individual paw (Stößel et al., 2017). Therefore, the rats sat within the staircase apparatuses on the plinth in the middle not being able to turn around and only reaching the left/right stairs with the respective paw. Each of the stairs is composed of seven steps, and was equipped with three sugar pellets each (AIN-76A Rodent Tablet 45 mg IRR, Lot number: 12SEP17RTD1, TestDiet^TM^, United States). After a 15 min testing period, remaining sugar pellets on the stairs and also sugar pellets on the ground (representing failed attempts) were summed up. Post-surgically, the test was performed at 4, 8, 12, and 16 weeks.

Forelimbs were classified as successfully participating as soon as more than three pellets were retrieved, because over time and with further slightly increasing body size, the animals could reach three pellets on the first step with their tongue and mouth.

### Non-invasive Electrodiagnostic Recordings – Evaluation of Thenar Muscle Reinnervation

Healthy baseline reference values of non-invasive electrodiagnostic measurements were recorded from the anesthetized animals, right before surgery. Post-surgical recordings were performed every fourth week.

Under deep anesthesia (body temperature was controlled and approximately 36.6 ± 0.3°C), animals were placed in supine position and single stimulating electric impulses (100 μs, 1 Hz) were induced by transcutaneous monopolar needle electrodes. Stimulation intensity was constantly increased up to 30% supramaximal level. The reconstructed median nerve was either stimulated proximal to the injury site in the axillary region or distal to the graft at the elbow. Evocable compound muscle action potentials (CMAPs) were recorded transcutaneously from the thenar muscles (Stößel et al., 2017). Despite possible co-stimulation of the ulnar nerve in the axillary region, CMAP recordings from the thenar muscles only result from the median nerve stimulation, because of the anatomical situation and trajectory of the median nerve (Stößel et al., 2017).

Semi-automatical evaluation of the amplitudes caused by the evocable CMAPs (manual setting of baseline to negative peak of M-wave) was performed by using a Dantec^^®^^ Keypoint^^®^^ Focus device (Natus Europe GmbH, Germany). If no evocable CMAP or CMAPs with no clear negative M-wave peak could be detected, a zero value was included into the statistical analysis.

### Nerve Immunohistochemistry in the Distal Nerve Grafts – Quantification of Axonal Profiles

At 16 weeks post-surgery, all animals were sacrificed in deep anesthesia in carbon dioxide atmosphere followed by cervical dislocation. The regenerated nerve tissue was removed from the lumen of the nerve guides, together with the central chitosan-film, in case of chitosan-film enhanced nerve guides. The tissue was fixed overnight [4% paraformaldehyde in phosphate-buffered saline (PBS, Dulbecco, Biochrom GmbH, Germany), 4°C, Sigma-Aldrich Chemie GmbH, Germany] before paraffin-embedding was performed.

From the distal portion of ANG grafts or at 3.7 mm proximal to the distal suture of tissue harvested from the nerve guides, series of 80 blind-coded cross-sections (7 μm) were prepared. Selected sections were Hematoxylin and Eosin (HE) stained in order to generate an overview of the composition of the regenerated tissue and to quantify median blood vessel areas (see the section “Quantification of median blood vessel area in the distal nerve grafts”). Consecutive sections were stained for neurofilament 200 (NF200) (Meyer et al., 2016a; Stenberg et al., 2017). For anti-NF200 staining, sections were incubated in blocking solution [3% milk powder (Bio Magermilch Pulver, Heirler Cenovis GmbH, Germany), 0.5% Triton-X 100 (Roche Diagnostics GmbH, Germany) in PBS] prior to incubation with primary rabbit anti-NF200 antibody (against phosphorylated NFeH, N4142, 1:500, Sigma-Aldrich GmbH, Germany, 4°C overnight) followed by incubation with Alexa-488-conjugated secondary goat anti-rabbit antibody (A11034, 1:1000, Invitrogen AG, Germany, 1 h at room temperature). The antibodies were diluted in blocking solution and the incubation steps separated by three-times washing in PBS. After nuclear counter staining with 4′, 6-diamidin-2-phenylindol (DAPI, 1:2000 in PBS, Sigma-Aldrich GmbH), sections were mounted using Mowiol (Calbiochem GmbH, Germany).

Representative photomicrographs of NF200-stained as well as HE-stained sections were created as multiple image alignments (MIA) using a BX51 microscope (Olympus GmbH, Germany).

Neurofilament 200-immunopositive axonal profiles were quantified with the help of ImageJ version 1.48 (National Institutes of Health, United States). Therefore, areas containing NF200-immunopositive axons were used to determine the regions of interest (ROI), which were needed to quantify the particle number later. By using a threshold strategy, NF200-photomicrographs were converted to binary images. Threshold pixel values reached from minimum 71 to maximum 172. This allowed distinguishing between individual nerve fibers. Afterwards NF200-immunopositive axonal profiles within the ROI were detected by using the particle analysis function of ImageJ. To avoid the detection of background noises, only particles with a size bigger than 0.001 Pixel^2^ were counted.

### Quantification of Median Blood Vessel Area in the Distal Nerve Grafts

Virtual slides of HE-stained sections were created with the help of macroscopic tissue slide scanner (Axio Scan.Z1, Carl Zeiss Microscopy GmbH, Germany). Tissue sections were scanned at 40× magnification. Cross sectional areas (μm^2^) of blood vessels within complete sections were quantified with the help of ZEN Imaging Software version 2.5, blue edition (Carl Zeiss Microscopy GmbH, Germany). Therefore, blood vessels within HE-photomicrographs were identified and their inner border, meaning the endothelial layer adjacent to the lumen, was contoured by using the Graphics tool and its area was quantified. Only blood vessels with visible erythrocytes within their lumen or, if no erythrocytes could be detected, with an intact endothelial layer were included into the evaluation.

### Nerve Histomorphometry Distal to the Nerve Grafts

Segments of the nerve, which were harvested directly distal to the nerve grafts, were fixed in Karnovsky solution, flushed in sucrose–sodium cacodylate buffer. Post-fixation was performed in 1% osmium tetroxide for 1.5 h before myelin sheaths were stained with 1% potassium dichromate and hematoxylin (Korte et al., 2011). Afterwards the samples were embedded into Epon and semi-thin cross-sections (1 μm) were prepared. To enhance the myelin staining a toluidine blue staining was performed. Finally, sections were mounted by using Mowiol.

Histomorphometrical evaluation was performed as described before (Stößel et al., 2017). Two sections of each reconstructed median nerve (*n* = 8 per group) were randomly selected and were evaluated at light microscopic level (BX50 microscope Olympus GmbH, Germany; 100× magnification) equipped with a prior controller (MBF Bioscience, United States). Total fiber numbers were determined using a two-dimensional procedure (optical fractionator; grid size: 150 × 150 μm^2^; counting frame size 30 × 30 μm^2^) by means of Stereo Investigator version 11.04 (MBF Bioscience). Therefore, systematic random sampling (Geuna, 2000) was applied to pick 12–16 sampling fields, depending on the size of the cross sectional area. In each of these sampling fields a two-dimensional dissector procedure was performed. Here, only the “tops” of fibers, meaning the first part of the fiber’s border that touched the edge of the counting frame, were counted to overcome the “edge effect” (Geuna, 2000).

In four photomicrographs (100× magnification) of each section, which were randomly selected, assessment of axon and fiber diameters and myelin thicknesses (G-ratio plug-in^1^, ImageJ version 1.48, National Institutes of Health, United States) was performed in, with an examination of 10 axons per picture, 80 axons per specimen, and 640 axons per group. Axon and fiber diameter were calculated based on the assumption of a circular shape.

### Statistical Analysis

Statistical analyses were applied to the data obtained in this study by using GraphPad Prism version 6.07 (GraphPad Software, United States). To detect significant differences, we resorted to two-way ANOVA followed by Tukey’s multiple comparisons and Kruskal–Wallis test followed by Dunn’s multiple comparisons. For statistical analysis of the qualitative outcome of the functional evaluation, we calculated the proportion of animals per group displaying evocable CMAPs or successful participation in either the grasping or the staircase test as percentages (0–100%) and compared them pair-wise with the Chi-square test. The *p*-value was set to *p* < 0.05 as significance level. All results are displayed as mean ± SEM or median ± range as indicated.

## Results

### Overall Qualitative Assessment of Functional Recovery

For full clarity, it needs to be stressed again that in this study 10 mm median nerve gaps were bilaterally reconstructed in 16 female Lewis rats and that the numbers given for specimen analyzed per group consequently refer to median nerves repaired with the respective approach (*n* = 8 for each group) instead of the number of animals studied.

Table 1 summarizes the qualitative results from the functional evaluation performed. For each evaluated parameter a more detailed description of the obtained results follows.

**Table 1:** Summary of functional recovery based on successful participation in the grasping and the staircase test and on evocable CMAPs recorded from the thenar muscle upon electric stimulation of the reconstructed median nerve.

		4 Weeks post-surgery		8 Weeks post-surgery		12 Weeks post-surgery		16 Weeks post-surgery		Total best	
		Forelimbs/group	[%]	Forelimbs/group	[%]	Forelimbs/group	[%]	Forelimbs/group	[%]	Forelimbs/group	[%]
Grasping test^a^	ANG	0/8	0.0	8/8	100.0^•^	6/8	75.0^#, 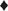 ^	6/8	75.0^#, 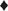 ^	8/8	100.0^•^
	CNG	0/8	0.0	1/8	12.5^ 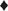 ,∗^	3/8	37.5	3/8	37.5	6/8	75.0
	CNG[F]	0/8	0.0	0/8	0.0	4/8	50.0	3/8	37.5	5/8	62.5
	corrCNG[F]	0/8	0.0	0/8	0.0	6/8	75.0^#, 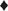 ^	5/8	62.5^#, 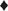 ^	7/8	87.5^ 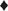 ^
Staircase test^b^	ANG	1/6	16.7^•^	6/6	100.0^•^	6/6	100.0^#, 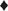 ^	6/6	100.0^#, 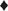 ^	6/6	100.0^#, 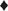 ^
	CNG	0/6	0.0	1/6	16.7	4/6	66.7^ 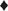 ^	4/6	66.7	4/6	66.7
	CNG[F]	0/6	0.0	1/6	16.7	3/6	50.0	5/6	83.3^#^	5/6	83.3^#^
	corrCNG[F]	0/6	0.0	1/6	16.7	6/6	100.0^#, 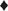 ^	6/6	100.0^#, 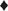 ^	6/6	100.0^#, 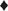 ^
Electro-diagnostic recordings^c^	ANG	8/8	100.0^ 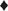 ,∗^	8/8	100.0^#, 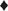 ^	8/8	100.0	8/8	100.0	8/8	100.0
	CNG	8/8	100.0^ 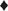 ,∗^	7/8	87.5	8/8	100.0	8/8	100.0	8/8	100.0
	CNG[F]	5/8	62.5	7/8	87.5	8/8	100.0	8/8	100.0	8/8	100.0
	corrCNG[F]	6/8	75.0	8/8	100.0^#, 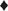 ^	8/8	100.0	8/8	100.0	8/8	100.0

*The process of recovery started with evocable CMAPs upon electrodiagnostic measurements, followed by participation in the staircase test and at latest participation in the grasping test was detected. At the end of the study, all animals showed recordable CMAPs. Full recovery of gross motor function (grasping test) in 100% of the tested forelimbs was only achieved by the ANG group, while full recovery of fine motor skills (staircase test) in all tested forelimbs was present in the ANG and corrCNG[F] groups. ANG, autologous nerve graft; CNG, chitosan nerve guide; CNG[F], chitosan-film enhanced chitosan nerve guide; corrCNG[F], corrugated chitosan-film enhanced chitosan nerve guide. Values are given as total numbers (forelimbs successfully participating per group) as well as percentages (%). ^a^Forelimbs were evaluated as successfully participating when displaying recovery of function of Category 3 (ability to grasp and pull the bar with a detectable force). ^b^Forelimbs were evaluated as successfully participating, when they retrieved more than three pellets because three pellets on the first step could be reached with their tongue and mouth. ^c^Forelimbs were evaluated as successfully, when evocable CMAPs recorded from the thenar muscle were detected. Statistical differences were calculated with the Chi-square test between single group pairs at the same time point (^•^p < 0.05 ANG vs. all nerve guide groups, ^#^p < 0.05 vs. CNG, 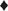
p < 0.05 vs. CNG[F], ^∗^p < 0.05 vs. corrCNG[F]).*

**FIGURE 1:**
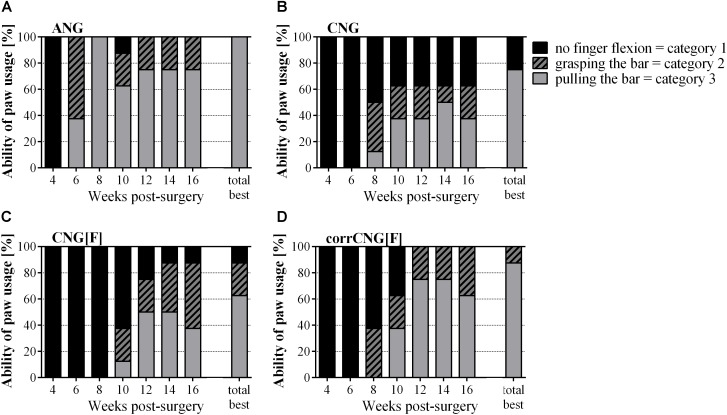
Percentage of the individual paw usage abilities based on video-recorded reflex-based grasping test performed every 2 weeks over 16 weeks post-surgery. Pulling the bar with a force is the most complete ability to be recovered (Category 3). This recovery of gross motor function is achieved by all forelimbs of the ANG group **(A)**. Among the artificial nerve guides corrCNG[F]s **(D)** led to the highest recovery rate. No statistical evaluation was applied. Values are given as percentages in relation to all evaluated forelimbs per group. **(A)** ANG, autologous nerve graft; **(B)** CNG, chitosan nerve guide; **(C)** CNG[F], chitosan-film enhanced chitosan nerve guide; **(D)** corrCNG[F], corrugated chitosan-film enhanced chitosan nerve guide: *n* = 8.

Forelimbs were evaluated as successfully participating in the grasping test when displaying recovery of function of category 3 (ability to grasp and pull the bar with detectable force, see also Figure 1). Forelimbs were evaluated as successfully participating in the staircase test when they retrieved more than three pellets, because the three pellets initially placed on the first step could be reached with their tongue and mouth. With regard to electrodiagnostic measurements forelimbs were evaluated as successful, when evocable CMAPs, recorded from the thenar muscle, were detected.

As depicted in Table 1, the electrodiagnostic measurements showed similar results for the ANG and corrCNG[F] group as early as 8 weeks post-surgery, while performance in the other groups was still significantly inferior (*p* < 0.05, Chi-square test). At 12 weeks post-surgery ANG and corrCNG[F] reconstructed forelimb groups showed the same level of performance in the grasping and the staircase test which was again significantly superior to the performance of the other groups evaluated (*p* < 0.05, Chi-square test). The total best performance could be ranked number 1 for ANG reconstructed forelimbs (100% in all three tests applied), number 2 for corrCNG[F] (100% in electrodiagnostic testing and skilled fore-limb reaching test, 87.5% in reflexed-based motor task), and number 3 for CNG and CNG[F].

### Grasping Test – Evaluation of Reflex-Based Paw-Usage Ability

The reflex-based grasping test (Figure 1) was categorized as described above [Category 1 – no finger flexion while touching the grasping bar; Category 2 – ability to grasp the bar (closing digits around the bar) but not to hold it while being slowly withdrawn; Category 3 – ability to grasp and pull the bar with a detectable force (gross motor skills)].

Generally, recovery of gross motor function was fastest in the ANG group achieving 100% recovery 8 weeks post-surgery. At 4 weeks post-surgery, no ANG-treated forelimb showed finger flexion while 2 weeks later all animals were able to grab the bar. At this time point, three out of eight forelimbs in the ANG group were able to apply a certain force to the grasping frame. At 8 weeks post-surgery, all ANG group forelimbs regained their gross motor function. Finger flexion (Category 2) was possible in week 8 by 37.5% (3/8) of forelimbs of the CNG and corrCNG[F] groups, while the first 12.5% (2/8) of forelimbs of the CNG[F] group showed finger flexion at 10 weeks post-surgery. At 12 weeks after surgical intervention, 37.5% of forelimbs in the CNG group, 50% (4/8) in the CNG[F], and 75% (6/8) of the forelimbs that received median nerve reconstruction with corrCNG[F] had recovered the ability to encompass the grasping bar, to close the digits around it and to pull is with some force (Category 3).

Over time, the animals showed less motivation to participate in this test, which induced little fluctuations in the performances from 8 weeks post-surgery onward. Therefore, the total best performance of each animal was considered additionally (Figure 1, see also Table 1).

Looking at the total best performances, none of the nerve guide groups achieved recovery of gross motor skills in all forelimbs in contrast to the ANG group. The ability to grasp and pull the bar with a recordable force (Category 3) recovered in 75% of the CNG group, whereby the residual 25% remained without finger flexion (Category 1). 62.5% of the CNG[F]-reconstructed forelimbs regained gross motor function. 25% of this group had the ability for finger flexion (Category 2), while one reconstructed forelimb (12.5%) remained without finger flexion (Category 1). Among the artificial nerve guide groups regeneration was most complete in the corrCNG[F] group (87.5%, Category 3). The residual 12.5% were able to flex their fingers (Category 2).

### Staircase Test – Evaluation of Skilled Forelimb Reaching Ability

Healthy baseline reference values for each individual paw, which were calculated as mean maximum number of pellets retrieved in the last 3 days of training, display that animals were able to retrieve a median of 7.84 pellets per paw at the end of the training period (data not shown). Eight forelimbs of five animals (left and right forelimbs of three animals, left forelimbs of two animals) had to be excluded from evaluation, since these animals only achieved a median of 0.84 retrieved pellets pre-surgically and their participation in the test did not improve post-surgically due to lack of motivation. This resulted in a number of *n* = 6 animals per group evaluated in the staircase test.

**FIGURE 2:**
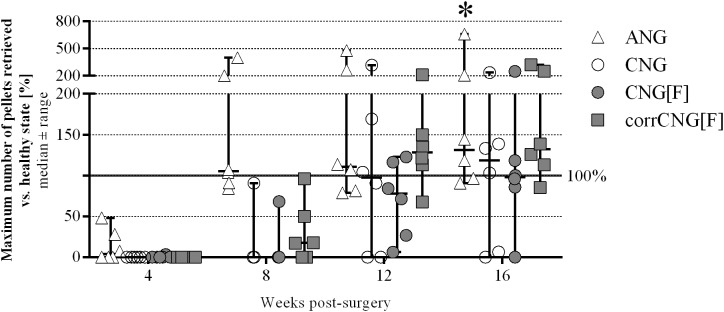
Quantitative results of the staircase test showing recovery of skilled forelimb reaching revealed by individual success rates in pellet retrieval over 16 weeks post-surgery. The success rates of ANG-reconstructed forelimbs significantly increased 16 weeks after reconstruction when compared to the 4-week time point. CorrCNG[F]-reconstructed forelimbs recovered to the same extend as forelimbs of the ANG group, while not all forelimbs of the CNG and CNG[F] groups achieved pre-surgical performance levels. Two-way ANOVA showed an effect of the parameters 4 vs. 16 weeks post-surgery [*F*(3,80) = 12.9, *p* < 0.0001] and groups [*F*(3,80) = 5.59, *p* = 0.0016] but there was no interaction. Tukey’s multiple comparisons were applied to detect significant differences (^∗^*p* < 0.05 vs. 4 weeks post-surgery within the same group). Values are displayed as median ± range and displayed as percentages in relation to pre-surgical healthy nerve mean values shown as baseline at 100%. ANG, autologous nerve graft; CNG, chitosan nerve guide; CNG[F], chitosan-film enhanced chitosan nerve guide; corrCNG[F], corrugated chitosan-film enhanced chitosan nerve guide: *n* = 6).

To exclude individual paw preference and behavioral influences, post-surgical outcomes are presented as percentages from healthy individual reference values, which were calculated as 100% (Figure 2).

At 4 weeks post-surgery two ANG-reconstructed forelimbs participated in the test, but only one of the two ANG-treated forelimbs participated successfully (retrieving more than three pellets, see also Table 1). At 8 weeks post-surgery one forelimb of each nerve guide group performed successfully (see also Table 1), whereby three additional forelimbs in the corrCNG[F] group started to participate. At this time point, all ANG-reconstructed forelimbs participated successfully (retrieving more than three pellets, individual success rates reached from 84.3 to 399.4%, median success rate: 105.5% of the maximum pellets retrieved in healthy state). At 12 weeks post-surgery, four forelimbs of the CNG group and three forelimbs of the CNG[F] group participated successfully and two additional animals of the CNG[F] group evidently started to participate. At the same time, all forelimbs of the corrCNG[F] group (median individual success rate: 128.1%) showed successful participation. While the number of paws that successfully participated in the staircase test did not further increase in the CNG group (4/6 forelimbs), one animal of the CNG[F] group was not able to reliably regain fine motor skills until 16 weeks after surgery. After 16 weeks of observation the median maximum number of pellets retrieved vs. healthy state exceeded the 100% healthy baseline in the ANG, CNG, and corrCNG[F] groups (ANG: 131.35; CNG: 118.25; corrCNG[F]: 132.10). Animals of the CNG group reached a median maximum number of 98.00% of healthy values. Despite the pre-surgical performance of the animals had reached a plateau after the 7 days training period, the finding, that healthy baseline reference values were far exceeded in part, can be attributed to the fact, that the process of learning was still ongoing during the observation period and not completed pre-surgically (Stößel et al., 2017). Also it cannot be excluded that the lesion and use of the temporarily impaired limb did recapitulate the learning process toward a better performance after recovery.

### Non-invasive Electrodiagnostic Recordings – Evaluation of Thenar Muscle Reinnervation

**FIGURE 3:**
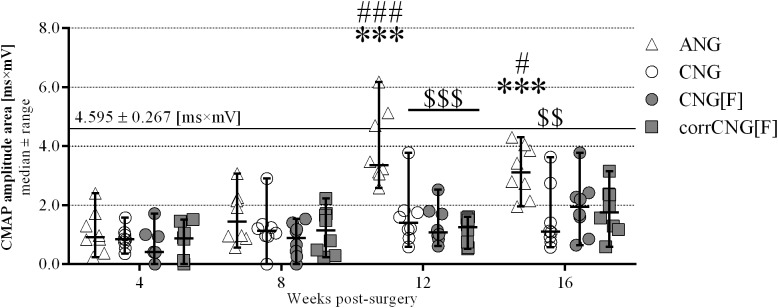
Evocable CMAPs amplitude areas as evaluated during electrodiagnostic recordings from the thenar muscle over 16 weeks observation period. ANG-reconstructed forelimbs showed significant improvement after 12 weeks post-surgery and significantly higher amplitudes compared to the nerve guide groups. Among the artificial nerve guide groups, no significant increase could be detected after the 16-week observation period. CNG-reconstructed forelimbs, however, revealed a significantly lower amplitude area in comparison to ANGs. The continuous horizontal line displays the pre-surgically recorded healthy nerve mean value (*n* = 32 forelimbs). Two-way ANOVA showed an effect of the parameters 12 vs. 4 and 8, as well as 16 vs. 4 and 8 weeks post-surgery [*F*(3, 112) = 19.35, *p* < 0.0001], groups [*F*(3,112) = 18.54, *p* < 0.0001), and the interaction between both parameters [*F*(9,12) = 3.534, *p* = 0.0007]. Tukey’s multiple comparisons were applied to detect significant differences (^∗∗∗^*p* < 0.001 vs. 4 weeks post-surgery within the same group; ^#^*p* < 0.05, ^###^*p* < 0.001 vs. 8 weeks post-surgery within the same group; ^$$^*p* < 0.01, ^$$$^*p* < 0.001 vs. ANG). Values are given as median ± range (*n* = 8). ANG, autologous nerve graft; CNG, chitosan nerve guide; CNG[F], chitosan-film enhanced chitosan nerve guide; corrCNG[F], corrugated chitosan-film enhanced chitosan nerve guide.

Compound muscle action potential amplitude areas resulting from stimulation distal to the graft were recorded in order to estimate the number of axons participating in thenar muscle reinnervation (Figure 3). Healthy baseline reference values for each paw were determined right before surgery (healthy mean: 4.595 ± 0.267 ms ^∗^ mV).

At 4 weeks, most reconstructed forelimbs of all groups showed evocable signals (ANG: 8/8, CNG: 8/8, CNG[F]: 5/8, corrCNG[F]: 6/8, see also Table 1). There were no significant differences in the CMAP amplitude areas when comparing all groups (two-way ANOVA). While CMAP amplitude area in the ANG group was highest (1.085 ms ^∗^ mV) it was smallest in the CNG[F] group (0.561 ms ^∗^ mV). In comparison to the 4-week time point, medians only slightly increased within the next 4 weeks in all groups (ANG: 1.595 ms ^∗^ mV, CNG: 1.208 ms ^∗^ mV, CNG[F]: 0.826 ms ^∗^ mV, corrCNG[F]: 1.111 ms ^∗^ mV). At that time point, stimulation in only one forelimb each in the CNG as well as in the CNG[F] group did not result in a recordable CMAP. In the ANG group, CMAP amplitude areas significantly increased at 12 weeks post-surgery (3.931 ms ^∗^ mV) when compared to 4 and 8 weeks post-surgery (*p* < 0.001, two-way ANOVA). At this time point, ANG reconstructed forelimbs delivered significantly higher CAMP amplitude areas than the artificial nerve guide groups (CNG: 1.601 ms ^∗^ mV, CNG[F]: 1.328 ms ^∗^ mV, corrCNG[F]:1.123 ms ^∗^ mV) with none of the forelimbs remaining unresponsive to stimulation (*p* < 0.001, two-way ANOVA). Sixteen weeks after surgical intervention, CMAP amplitude areas of the CNG[F] and the corrCNG[F] group further increased while CNG reconstructed forelimbs still performed significantly less compared to the ANG group (*p* < 0.01, two-way ANOVA).

### Macroscopic Evaluation of the Regenerated Tissue Upon Explantation

**FIGURE 4:**
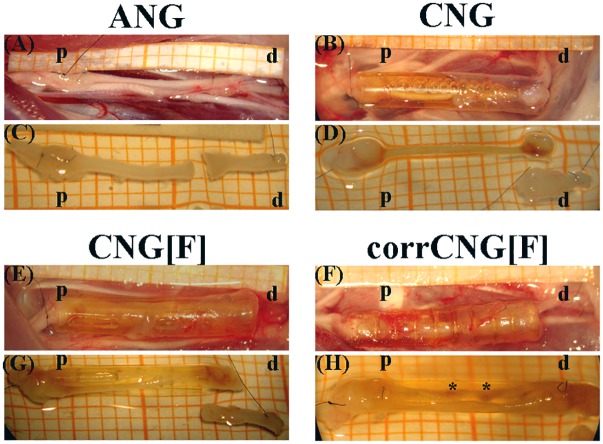
Representative pictures of the macroscopic appearance of the sutured grafts right before explantation **(A, B, E, F)** and of the explanted regenerated tissue at 16 weeks post-surgery **(C, D, G, H)**. For treatment of 10 mm median nerve gaps either ANGs **(A, C)**, hollow standard CNGs **(B, D)**, CNG[F]s **(E, G)**, or corrCNG[F]s **(F, H)** were inserted and sutured at the corresponding nerve end (p = proximal, d = distal). In ANG- and CNG-reconstructed forelimbs one nerve strand was formed. On the other hand, CNG[F]s and corrCNG[F]s led to two thinner nerve strands, generally divided from each other by the z-folded chitosan-film. Perforations within the chitosan-films allowed for growth of macroscopically visible, eventually blood supplied, connections between the two nerve strands (indicated by ^∗^).

To generate an overview of tissue regeneration between the proximal and the distal nerve end, ANGs as well as regrown tissue inside the diverse nerve guides were first surveyed macroscopically at 16 weeks post-surgery (Figure 4). Reconstruction of the nerves with ANGs and corrCNG[F]s resulted in full-distance regenerated tissue in all animals of the group. Two reconstructed median nerves of the CNG group revealed no connections between the nerve ends and in one graft only a very thin connection was detectable, which was too thin for paraffin embedding. For the latter sample the nerve distal to the graft was, however, processed for nerve morphometry (see the section “Quantification of blood vessel area in the distal chitosan nerve grafts”). Also, implantation of CNG[F]s led to one forelimb without any regenerated tissue.

While reconstruction with ANGs and CNGs, respectively, led to one thick connection between the proximal and distal nerve ends, reconstruction with either type of CNG[F]s or corrCNG[F] resulted in the formation of two thinner tissue cables, one on each side of the film, connecting the two nerve ends. At the level of chitosan-film perforations, tissue which grew through the perforations could be detected. These tissue bridges formed a connection between the two regenerated tissue cables.

### Nerve Immunohistochemistry in the Distal Nerve Grafts – Quantification of Axon Profiles

**FIGURE 5:**
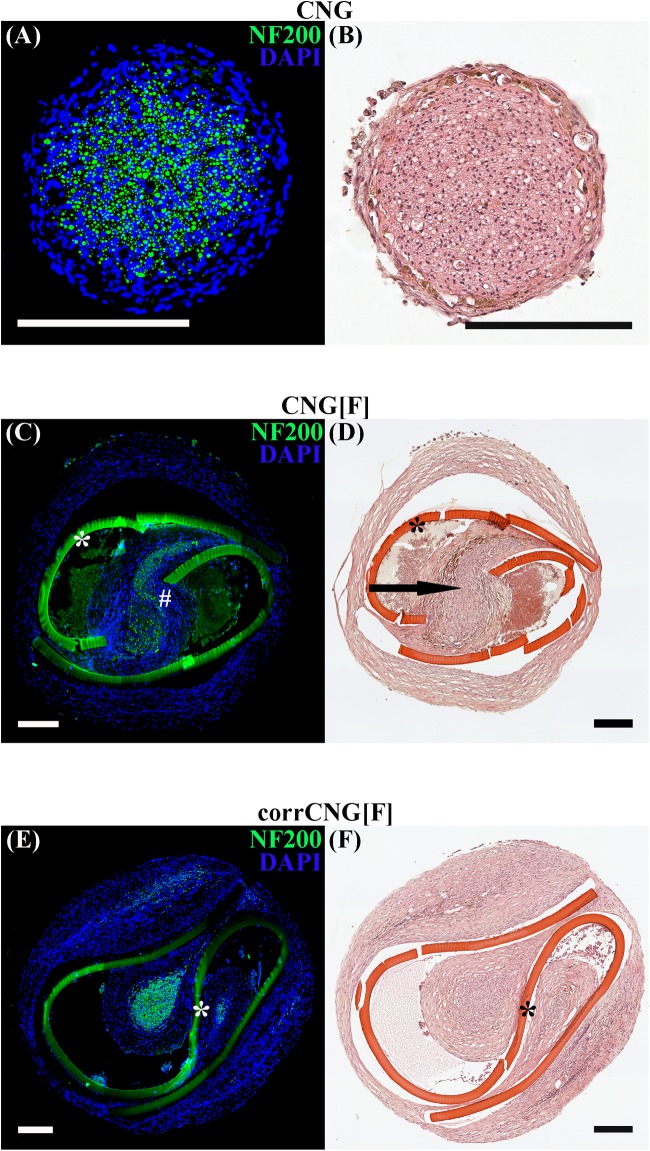
Representative photomicrographs of consecutive cross-sections through the regenerated tissue within the distal nerve graft at 16 weeks post-surgery. Immunohistological staining **(A, C, E)** against NF200 (green) and DAPI nuclear counter staining (blue) display immunodetection of all regenerated axonal profiles. HE staining **(B, D, F)** displays an overview of the composition of the regenerated tissue. Thicker and single-strand tissue connections between proximal and distal nerve ends were found in the ANG and CNG groups. Axonal staining proofed that on each side of the chitosan-film the regenerated tissue contained NF200-positive axons. Arrow is indicating the tissue bridge, which was formed inside the chitosan-film perforation and connected the two nerve cables (chitosan-film indicated by ^∗^, NF200-positive axonal profiles within the tissue bridge indicated by #). CNG, standard chitosan nerve guide; CNG[F], chitosan-film enhanced CNG; corrCNG[F], corrugated chitosan-film enhanced chitosan nerve guide.

For histological evaluation of the regenerated tissue within the nerve grafts, not all samples could be used. This was related to very thin or even missing connections between the proximal and distal nerve ends, so that no tissue could be processed for immunocytochemistry. Therefore, regenerated tissue from *n* = 8 ANG- and corrCNG[F]-treated forelimbs, *n* = 5 CNG-treated forelimbs, and *n* = 7 CNG[F]-treated forelimbs were incorporated into the evaluation. Cross-sections were prepared at the distal end of the ANG grafts and at 3.7 mm proximal to the distal suture site within the nerve grafts. These cross-sections were stained for HE to demonstrate the composition of the regenerated tissue (Figure 5B,D,F) and to analyze the area of blood vessel within the regenerated tissue inside the nerve guides (see the section “Nerve immunohistochemistry in the distal nerve grafts – quantification of axon profiles”).

**FIGURE 6:**
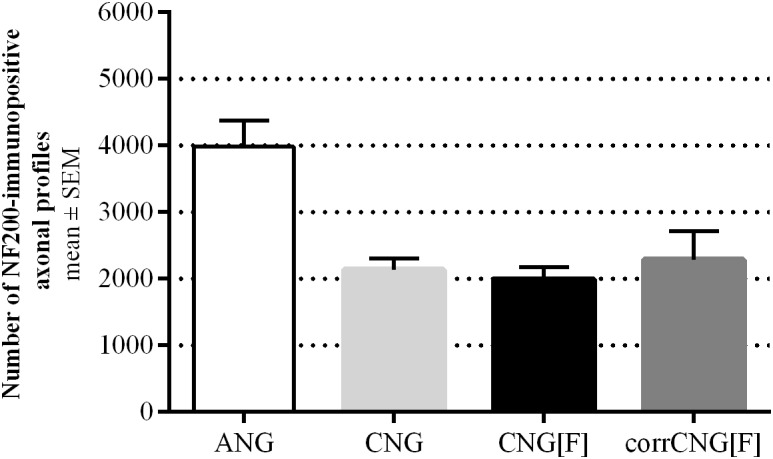
Quantification of NF200-immunopositive axonal profiles at midgraft-level at 16 weeks post-surgery. ANG reconstructions revealed the highest numbers of NF200-positive axonal profiles among all tested implants. CorrCNG[F]s showed slightly increased numbers of immunopositive axonal profiles, when compared to the other artificial nerve guides tested. Kruskal–Wallis test [*H*(3, *N* = 28) = 7.74, *p* = 0.0517] followed by Dunn’s multiple comparisons were applied. No significant differences were detected. Values are given as mean ± SEM. ANG, autologous nerve graft; corrCNG[F], corrugated chitosan-film enhanced chitosan nerve guide: *n* = 8; CNG, chitosan nerve guide: *n* = 5; CNG[F], chitosan-film enhanced chitosan nerve guide: *n* = 7.

Hematoxylin and Eosin-stained cross-sections show single strands of regenerated tissue in the ANG and CNG (Figure 5B) groups, while two strands, one at each side of the chitosan-film, were formed in the CNG[F] (Figure 5D) and corrCNG[F] (Figure 5E) groups. HE stained sections eventually also illustrated, that film-perforations contained regrown tissue as well (Figure 5F, indicated by arrow). Consecutive sections of those, that underwent HE staining, were stained for NF200 and counter-stained with DAPI. This enabled the immunodetection of regenerated axonal profiles in the distal nerve grafts. Representative photomicrographs (Figure 5A,C,E) show the presence of NF200-immunopositive axons within the regenerated tissue strands. In chitosan-film enhanced nerve guides, axonal profiles were detectable at both sides of the chitosan-film (Figure 5C,D, indicated by ^∗^), but eventually also in tissue bridges connecting the two nerve strands (Figure 5C, indicated by #). As ANGs and CNGs led to regeneration of thicker tissue cables, we further investigated if this was associated with a higher number of regrown axons within the distal graft in comparison to CNG[F]s and corrCNG[F]s. Therefore, quantification of NF200-immunopositive axonal profiles was performed (Figure 6). Median nerve reconstruction with ANGs revealed the highest number of NF200-immunopositive axonal profiles (3981 ± 393.9). Numbers of NF200-immunopositive axonal profiles were smaller in the distal CNGs (2139.1 ± 165.5), CNG[F]s (1993.4 ± 185.4), and corrCNG[F]s (2285.2 ± 425.2), but no significant differences could be detected between all groups (Kruskal–Wallis test). Among the bioartificial nerve guide groups, the highest number of NF200-immunopositive axonal profiles was detected in distal nerve graft samples from corrCNG[F] reconstructed forelimbs.

### Quantification of Blood Vessel Area in the Distal Chitosan Nerve Grafts

**FIGURE 7:**
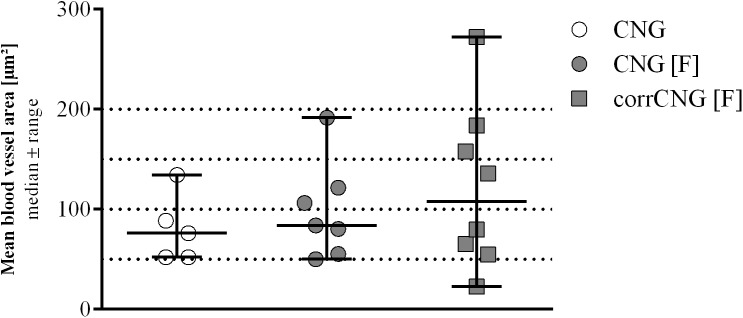
Quantitative results of the mean blood vessel area within the distal nerve graft at 16 weeks post-surgery. No significant differences could be detected among the experimental groups. Kruskal–Wallis test [*H*(2, *N* = 20) = 0.929, *p* = 0.6456) followed by Dunn’s multiple comparisons were applied to detect significant differences. Values are given as median ± range. CNG, chitosan nerve guide: *n* = 5; CNG[F], chitosan-film enhanced chitosan nerve guide: *n* = 7; corrCNG[F], corrugated chitosan-film enhanced chitosan nerve guide: *n* = 8.

In previous studies we have postulated that introducing the chitosan-films into the CNGs may increase the vascularization of the regenerated tissue (Stenberg et al., 2017; Stößel et al., 2018b). To analyze this in some more detail, we quantified the mean area of clearly identifiable blood vessels in the HE sections obtained from the CNG groups (*n* = 5 CNG, *n* = 7 CNG[F], and *n* = 8 corrCNG[F]). As depicted in Figure 7, the evaluation of the mean blood vessel area did not reveal any significant differences between the experimental groups (Kruskal–Wallis test). The data point out, however, that larger mean blood vessel areas (e.g., >100 μm^2^) have a higher probability to be formed when CNGs contain a chitosan-film.

### Nerve Histomorphometry Distal to the Nerve Grafts

**FIGURE 8:**
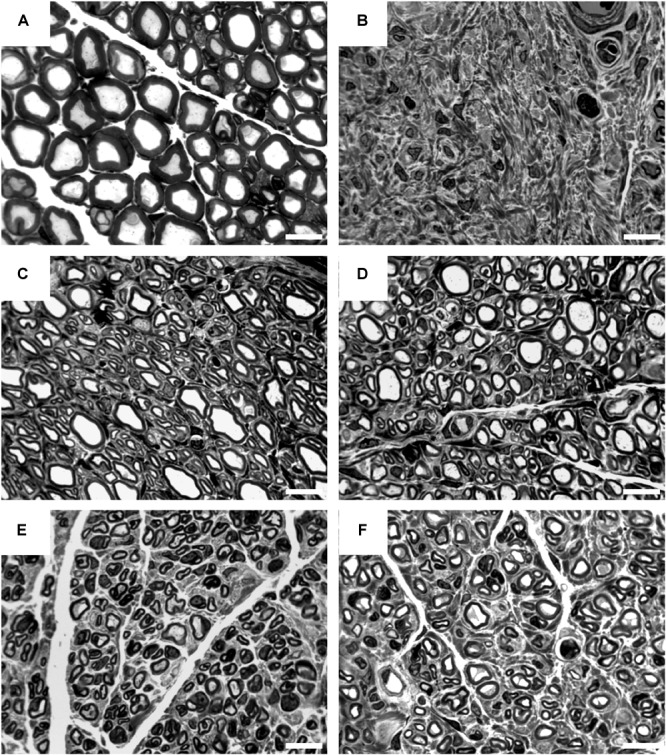
Representative pictures of toluidine blue-stained semi-thin cross-sections of distal nerve segments at 16 weeks post-surgery. Images show healthy nerve segments **(A)** serving as control compared to distal nerve segments of reconstructed median nerves **(B–F)**. **(B)** Example of no axonal regeneration from the CNG[F] group. Examples of regenerated nerve samples from the ANG group **(C)**, CNG group **(D)**, CNG[F] group **(E)**, and corrCNG[F] group **(F)**. White scale bars display 10 μm.

For stereological and morphometrical assessment of regenerated myelinated axons, semi-thin cross-sections were prepared from distal nerve segments of reconstructed median nerves at 16 weeks post-surgery (Figure 8). Two samples of the CNG group with no evident regrown tissue detected during macroscopic inspection (see the section “Non-invasive electrodiagnostic recordings – evaluation of thenar muscle reinnervation”) were excluded from evaluation (*n* = 6). In the CNG[F] group also one sample was excluded due to lack of regeneration (*n* = 7).

**FIGURE 9:**
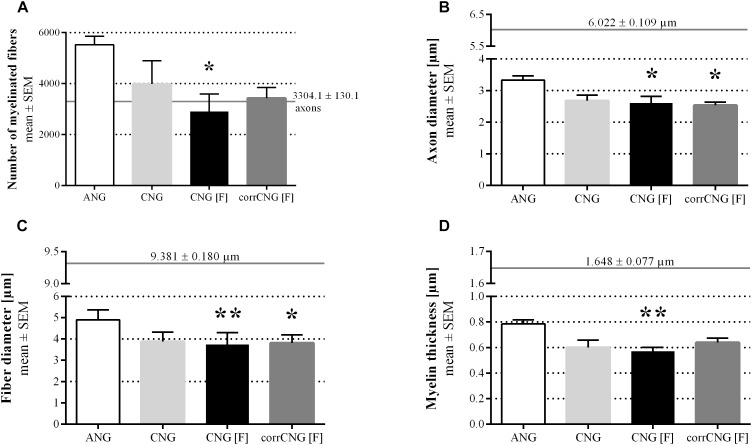
Quantitative results of the nerve morphometrical analyses of distal nerve segments of reconstructed median nerves at 16 weeks post-surgery. Bar graphs are representing the total numbers of myelinated fibers **(A)**, axon diameters **(B)**, fiber diameters **(C)**, and myelin thicknesses **(D)**. Samples from ANG-reconstructed forelimbs were superior to the artificial nerve guides concerning all tested parameters. Only single events of statistically significant differences could be detected. CNG[F]s and corrCNG[F]s led to significantly lower axon diameters when compared to the ANG group. Additionally, CNG[F]s led to significantly thinner nerve fibers and myelin sheaths when compared to samples from ANG-reconstructed forelimbs. Horizontal continuous lines display healthy nerve mean values (*n* = 6). Kruskal–Wallis test [**(A)** *H*(3, *N* = 29) = 10.19; **(B)** *H*(3, *N* = 29) = 11.24; **(C)** *H*(3, *N* = 29) = 15.03; **(D)** *H*(3, *N* = 29) = 12.34] with Dunn’s multiple comparisons were applied to detect significant differences (^∗^*p* < 0.05, ^∗∗^*p* < 0.01 vs. ANG at the same time point). Values are given as mean ± SEM. ANG, autologous nerve graft; corrCNG[F], corrugated chitosan-film enhanced chitosan nerve guide: *n* = 8; CNG, chitosan nerve guide: *n* = 6; CNG[F], chitosan-film enhanced chitosan nerve guide: *n* = 7.

Regarding the numbers of myelinated fibers (Figure 9A), axon diameters (Figure 9B), fiber diameters (Figure 9C), and myelin thicknesses (Figure 9D), samples resulting from ANG reconstructed forelimbs were superior over all bioartificial nerve guide groups tested. While no significant differences could be detected when comparing numbers of myelinated fibers of ANGs with CNGs and corrCNG[F]s, CNG[F]s revealed significantly lower numbers of myelinated fibers (*p* < 0.05, Kruskal–Wallis test). When looking at the axon diameters and fiber diameters, CNG(F)s and corrCNG[F]s showed significantly smaller diameters in both parameters when compared to the ANG group (*p* < 0.05 for axon diameter of CNG[F], corrCNG[F], and fiber diameter of corrCNG[F], *p* < 0.01 for fiber diameter of CNG[F], Kruskal–Wallis test). However, only CNG[F]s showed significantly thinner myelin sheaths when compared to ANGs (*p* < 0.01, Kruskal–Wallis test), whereas reconstruction with corrCNG[F]s revealed the thickest myelin sheaths among the nerve guide groups. Major variations or significant differences in g-ratio values (data not shown), however, were not detected among all groups (ANG: 0.67 ± 0.01; CNG: 0.68 ± 0.03; CNG[F] 0.68 ± 0.02; corrCNG[F] 0.66 ± 0.01).

## Discussion

In connection with peripheral nerve injuries the application of ANGs still represents the gold standard (Daly et al., 2012). As this method goes along with various downsides (Siemionow et al., 2010; Hallgren et al., 2013; Kuffler, 2014), researchers still try to substitute or replace the use of ANGs by alternative bioartificial nerve guide-based treatment strategies (Gu et al., 2014; Faroni et al., 2015).

For studying the properties of novel bioartificial nerve grafts, nerve transection models are commonly used. Complete nerve transection injuries are characterized by structural changes, including axonal and myelin breakdown (Deumens et al., 2010). As such the model has distinct differences to other peripheral nerve injury models, e.g., loose ligation models of the rat sciatic nerve. Nerve ligation models are studied in the context of the development of neuropathic pain in which early events at the lesion sites are characterized by edema and inflammatory infiltration (e.g., Pacini et al., 2010; Di Cesare Mannelli et al., 2016). One leading event after nerve transection injury, besides axonal and myelin breakdown, is also recruitment of macrophages that help in myelin clearance during Wallerian degeneration over 3–6 weeks after the injury (Gaudet et al., 2011; Faroni et al., 2015; Jessen and Mirsky, 2016). Since morphological changes after median nerve transection injury have been described elsewhere before (e.g., Ronchi et al., 2016), we were not focusing on these events in the current study. Here, besides thorough functional analyses, we focused on end-point histological analysis of the regenerated nerve tissue after 16 weeks post-surgery. At this late time point, edema or continued inflammatory infiltrations would only be expected in case of immune responses against the artificial nerve grafts or their degradation products. This was, however, never detected in other studies evaluating chitosan-based nerve guides before (e.g., Haastert-Talini et al., 2013; Stößel et al., 2018a,b). We further did not study effects of any complementary treatment to protect peripheral nerve arrangement and to prevent development of neuropathic pain, like, e.g., daily oral administration of rosemary extracts (Pacini et al., 2010; Di Cesare Mannelli et al., 2016), because the rat median nerve transection and repair model has not been reported before to induce neuropathic pain.

In this study we compared ANGs and three different types of chitosan-based nerve guide grafts with regard to their support for peripheral nerve regeneration after acute repair of 10 mm median nerve defects. The nerve guides evaluated consisted of standard hollow CNGs (Haastert-Talini et al., 2013), CNG[F]s ( Meyer et al., 2016a), or corrCNG[F]s. The corrugated structure of the outer wall of hollow CNGs is thought to make them most suitable for bridging peripheral nerve gaps in highly mobile locations (Stößel et al., 2018b). Highly mobile locations are for example human digits, in which digital nerves, originating from the median nerve, are even traveling across joints. Previous studies have demonstrated that by inserting a z-folded chitosan-film and creating a two-chambered chitosan nerve guide (CNG[F]), the support of functional recovery after immediate and delayed critical defect length rat sciatic nerve repair could even be increased in comparison to standard CNGs (Meyer et al., 2016a; Stenberg et al., 2017). Consequently, in this current study, we have evaluated corrCNG[F]s for their regeneration supporting properties in the rat median nerve model. We suppose the rat median nerve model to be more translational than the classic rat sciatic nerve model (Stößel et al., 2018b) with regard to the repair of human digital nerve injuries, which are displaying the majority of clinically relevant peripheral nerve injuries (McAllister et al., 1996; Renner et al., 2004).

Clinically most important for patients suffering from peripheral nerve injury is the recovery of fine and gross motor function (Fugleholm et al., 2000; Valero-Cabre et al., 2001), which can still not be guaranteed even after nerve reconstruction with the use of ANGs (Deumens et al., 2010; Isaacs, 2010). The median nerve model used in the current study allows to define rather precisely the onset of different level motor functional recovery and to evaluate the final recovery level of the different motor functions (Stößel et al., 2017). For a good comparison of results and like in many other studies in the field and in accordance to our previous work (Stößel et al., 2017) we used female young adult rats (15 weeks at beginning of adaptation to test conditions) in the current study.

Our results demonstrate that median nerve reconstruction in the rat with corrCNG[F]s achieved functional results comparable to the results obtained after reconstruction with ANGs. Even though the functional recovery was fastest and most complete (100% recovery rate) in the ANG group already 8 weeks post-surgery, corrCNG[F]s significantly accelerated the recovery of fine and gross motor functions when compared to CNGs and CNG[F]s. Final recovery levels for skilled forelimb reaching ability were even significantly superior in the corrCNG[F] group when compared to the two other nerve guide groups evaluated.

While this overall conclusion could easily be drawn from the results presented, this discussion will focus on a more detailed analysis of the evaluation methods used and the particular results they provided.

The reflex-based grasping test, which is used to analyze the recovery of gross motor function (Tupper and Wallace, 1980), was performed every second week in the current study. This is the simplest test in our test battery and certainly the less stress-full for the animals, because it does not require any specific preparation and is performed with the awaken animal. Therefore, in our preceding study (Stößel et al., 2017), the grasping test was performed weekly and was found to thereby discover rather precisely the onset of reflex-based gross motor functional recovery. But the weekly testing procedure also resulted in a reduced motivation of the animals to participate in the test (Stößel et al., 2017). This observation has also been described before (Bertelli and Mira, 1995), and therefore, to keep motivation to participate, we decided to perform the test only every second week in the current study. Still we were facing minor undulation in the recovery rate of gross motor function (ANG group between 8 and 10 weeks post-surgery; and either nerve guide groups between 14 and 16 weeks post-surgery), but were also able to detect differences in the recovery time and recovery rates among the groups. The grasping test confirmed that ANG reconstruction was most supportive for recovery of gross motor function, since exclusively the ANG group showed a 100% recovery rate in this test. Comparing the total best results among the nerve guide groups, use of corrCNG[F]s for reconstruction led to the highest percentage of forelimbs (87.5%) being able to apply a certain force to the bar (Category 3), which is the most complete ability to be recovered (Stößel et al., 2017). Notably, superiority of the corrCNG[F] grafts over the other nerve guides is also indicated by the observation, that none of the corrCNG[F] reconstructed forelimbs remained without ability of finger flexion (Category 2) in contrast to the CNG group (two animals without finger flexion) and the CNG[F] group (one animal without finger flexion).

The staircase test was performed for comprehensive evaluation of recovery of fine motor skills (Montoya et al., 1991). The test requires a preparatory period of restrictive feeding and can therefore only be applied on a monthly basis. The power of this test is again highly affected by the motivation of the animals to participate (Nikkhah et al., 1998; Galtrey and Fawcett, 2007). While in our previous study only 1 out of 16 animals demonstrated un-willingness to participate already during the pre-surgical habituation and training period (Stößel et al., 2017), in the current study, three animals were not at all sufficiently participating and two additional animals were only participating with their right forelimb. This situation was reducing the power of the test since it reduced the evaluated group size from initially eight to only six animals. Lewis rats had been described before to probably display less motivation to participate, but to also provide a potentially reduced learning ability (Nikkhah et al., 1998; Galtrey and Fawcett, 2007). On the other hand, some of the remaining animals in all evaluated groups achieved success rates highly above their pre-surgical values during the recovery period. Thus, this displays an ongoing process of learning after pre-surgical training and during the post-surgical observation period (Stößel et al., 2017). Although there is certainly more to consider when applying the staircase test paradigm for median nerve injury and repair studies, we again found it useful in depicting another type of motor functional recovery than the grasping test and in elucidating differences among our evaluated groups. Complete recovery of fine motor skills (100% recovery rate) was achieved in the ANG as well as by the corrCNG[F] group, while not all forelimbs of the CNG and CNG[F] groups achieved pre-surgical performance levels and some of them did not recover skilled forelimb reaching abilities at all.

When assembling the overall motor abilities recovered as assessed by us in the advanced rat median nerve model, one could even detect some striking results in single animals that need a more reflected view on the underlying axonal regeneration. One of the ANG-reconstructed forelimbs successfully participated in the staircase test already 4 weeks post-surgery (retrieving more than three pellets), while demonstrating no finger flexion in the grasping test at the same time. The same phenomenon also occurred in one animal of the CNG[F] group at 8 weeks post-surgery. Looking at these results one should assume that axons were correctly directed to the target muscles, since fine motor skills require accurate reinnervation (Galtrey and Fawcett, 2007). Furthermore, successful pellet retrieval requires the ability for finger flexion, resulting in the assumption that the absence of finger flexion in the grasping test may be again explained by a lack of motivation of the animals (Nikkhah et al., 1998; Galtrey and Fawcett, 2007). This observation can obviously not be related to the nerve graft applied for reconstruction and has therefore negatively biased the results from the grasping test. One should also consider that even a certain number of misdirected axons would lead to the ability for reflexed-based gross motor grasping function, because for this the amount of reinnervating axons is more important than the accuracy of reinnervation (Galtrey and Fawcett, 2007).

The previous considerations lead over to the discussion of the electrodiagnostic measurements applied in the current study. Similar to the staircase test, these measurements were performed on a monthly basis, because we have been experiencing low tolerability of repetitive anesthesia in a previous study (Korte et al., 2011). Also, the obtainable data by recording and analyzing evocable CMAP amplitude areas are providing a quantitative estimation on the degree of motor reinnervation, but no indication on its consequences on forelimb usage abilities. In our previous study we demonstrated that electrodiagnostic recordings from the thenar muscles are a valuable tool to elucidate the early onset of motor axon regeneration, which is somehow predictive for the time period needed until motor skills will also start to return (Stößel et al., 2017). We had a similar observation in the current study. At 4 weeks post-surgery, evoked CMAPs were recordable in 100% of ANG and CNG reconstructed forelimbs when still no finger flexion was detected with the grasping test and only one ANG-forelimb and none CNG-forelimb successfully participated in the staircase test. The same findings could be detected in the two other groups where the majority of the reconstructed nerves (CNG[F]: 62.5%; corrCNG[F]: 75.0%) transmitted evocable CMAPs to the thenar muscles, but none of the forelimbs achieved Category 2 (ability for finger flexion) in the grasping test or successfully participated in the staircase test. Occurrence of 100% of reconstructed forelimbs showing evocable thenar muscle CMAPs revealed a timeline among the groups with the ANG-group and CNG-group being the first (4 weeks post-surgery), the corrCNG[F] group the second (8 weeks post-surgery), and the CNG[F] group followed 12 weeks post-surgery.

Electrodiagnostic recordings provide a twofold estimation of functional recovery. First, the pure evidence of muscle reinnervation as detected by recording of evoked CMAPs, and second, the estimation of the quality of reinnervation as it can be retrieved from analyzing the CMAP amplitude area. The CMAP amplitude area correlates with the number of functioning axons (Cuddon, 2002). Although, in the current study, the CNG group showed the earliest onset of motor reinnervation among the bioartificial nerve guide groups, the CMAP amplitude area was still significantly smaller after 16 weeks post-surgery when compared to the ANG group, which achieved the overall highest median amplitude area among all nerve grafts tested. At this time point median CMAP amplitude areas were not anymore significantly different to the ANG values in the CNG[F] and corrCNG[F] groups.

Extrapolating these results now to usage abilities and evidently recovered motor skills is, however, also not fully possible. Although the median CMAP amplitude area recorded in the CNG[F]s ranged slightly above that one recorded from the corrCNG[F] reconstructed forelimbs, motor skills returned to a higher rate in the second group. This indicates again that CMAP amplitude recovery is not necessarily accompanied by precise regeneration of all motor axons (Archibald et al., 1991; Fugleholm et al., 2000; Valero-Cabre et al., 2001; Navarro and Udina, 2009; Pfister et al., 2011). Additionally, it demonstrates that also stimulation of finally misdirected axons contributes to the value of CMAP amplitude areas and these axons do not compulsorily lead to regeneration of especially fine motor skills (Galtrey and Fawcett, 2007).

Those electrodiagnostic measurements, as performed in our current study, should only be one tool to evaluate functional recovery of the rat median nerve, as must also be concluded from the macroscopic inspection of the nerve grafts upon explantation and the subsequent histomorphometrical analysis. The latter is also irreplaceable for a comprehensive evaluation of nerve repair approaches.

Upon explantation of the nerve grafts, we could macroscopically detect visible tissue connections between the proximal and the distal nerve end in all ANG and corrCNG[F] grafts, while surprisingly two nerve guides from the CNG group and one from the CNG[F] group did not contain sufficient amount of tissue. Although during establishment of the advanced median nerve model we did not find evidences that false positive recordings could occur from electrodiagnostic measurements (Stößel et al., 2017), we need to reconsider this possibility. In the current study, all forelimbs of the CNG and the CNG[F] group presented with evocable CMAPs in electrodiagnostic evaluation. Analysis of CMAP amplitude areas could again be judged to be of major value, since we at least detected that the false positive CMAPs displayed an area below the group median and histomorphometric analysis of distal nerve segments did not detect regrown axons.

Histomorphometrical analysis finally serves not only to reveal more quantitative data indicative also for the quality of axonal regeneration, but could also provide insight into tissue compositions related to good functional recovery.

As observed in previous studies (Meyer et al., 2016a; Stenberg et al., 2017), tissue regeneration through CNG[F]s results in growth of two nerve strands separated by the chitosan-film. As we have shown previously and here again, these nerve strands are eventually connected via tissue bridges that have formed within the perforations in the film. In the current study we were even able to detect NF200-immunopositive axons inside these tissue bridges.

Quantification of the number of NF200-immunopositive axonal profiles within the distal grafts revealed no significant differences within the groups, although the highest mean value was detected in the ANG group and the corrCNG[F] group showed slightly higher values than the two other nerve guide groups. These findings already correlate to our results from the motor skills analyses. We have previously postulated that chitosan-film enhanced nerve guides would attract a higher degree of neovascularization and that this may directly correlate to better functional outcome (Meyer et al., 2016a; Stenberg et al., 2017). Early revascularization is an important factor for successful regeneration as it is supposed to prevent apoptosis of Schwann cells, fibrosis, and failure of regeneration (Penkert et al., 1988). In addition, blood-derived macrophages are known to support peripheral nerve regeneration by producing growth factors and adhesion molecules (Fansa et al., 2001; Haastert-Talini et al., 2013; Mokarram et al., 2017; Stenberg et al., 2017). In the current study we determined the median blood vessel area in the distal nerve guides in order to elucidate potential differences in neovascularization among the different nerve guide types. And indeed, although not significant again, in the corrCNG[F] group, with the best functional outcome among the nerve guide groups, we found in 50% of the samples mean blood vessel areas above the group mean, while in the CNG[F] group and CNG group these are only 43 and 40%, respectively.

It is noteworthy that immunohistological evaluation of the number of NF200-immunopositive axonal profiles in the distal grafts resulted in reduced numbers of detected axons in comparison to the histomorphometrical analysis performed distal to the grafts. Among all investigated groups, more regenerated and myelinated nerve fibers were detected in the nerve specimen distal to the grafts than within the grafts. This could be found to be in contradiction to the assumption that not all nerve fibers that sprout proximally should reach their distal target and that the number of detectable axons will therefore get smaller at more distal locations. One should, however, consider that immunohistological staining and quantification in fluorescence microscopy may miss a certain amount especially of small regenerated axons, due to thresholds that have to be set manually. Therefore, a more reliable axon count will always result from nerve morphometrical analysis.

Nerve morphometry finally performed in specimen harvested distal to the grafts revealed that less axons regenerated in the nerve guide groups than in the ANG group, with CNG[F] samples showing significantly less axons. As shown before (Sinis et al., 2005; Haastert-Talini et al., 2013; Ronchi et al., 2017; Stößel et al., 2017), ANG reconstruction results in higher numbers of myelinated fibers in comparison to the artificial nerve guides and even compared to the healthy nerve. Healthy nerve values for numbers of myelinated fibers were exceeded by the ANG, CNG, and corrCNG[F] group, a phenomenon that has been described before in different models (Meyer et al., 2016a; Stenberg et al., 2017; Stößel et al., 2017). Probably the second most indicative nerve morphometrical parameter for functional recovery, which has been analyzed in the current study, is the myelin thickness. The myelin thickness determines the nerve conduction velocity (Valero-Cabre and Navarro, 2002; Korte et al., 2011) and could therefore have a direct impact on fine motor reaching skills. Axons from CNG[F] group samples displayed significantly thinner myelin sheaths than those from the ANG group, while the other nerve guide groups showed no significant differences. Samples from the corrCNG[F] group displayed slightly thicker myelin sheaths of their axons among the bioartificial nerve graft groups. These findings underline that corrCNG[F] supported functional regeneration of the reconstructed median nerve to a higher extend than the other CNGs evaluated.

In conclusion, we demonstrated that the use of corrCNG[F]s represents a promising approach for reconstruction of small nerves in a mobile extremity location. The results of the current study are translational for the repair of digital nerves in humans since different nerve guides were comprehensively tested against the gold standard ANG and the clinically approved classic hollow CNG.

## Ethics Statement

This study was conducted in accordance with the German animal protection law and with the European Communities Council Directive 2010/63/EU for the protection of animals used for experimental purposes. All experiments were approved by the Local Institutional Animal Care and Research Advisory Committee and permitted by the local authority (Lower Saxony State Office for Consumer Protection, Food Safety, and Animal Welfare Service, 33.12-42502-04-15/1761).

## Author Contributions

KH-T and TF contributed conception and design of the study. MF and KH-T conducted the experiments. OH produced the CNGs. MF and ND evaluated the functional recovery. MF organized the respective data presentation and performed the statistical data analysis. ND and JL performed the histomorphometrical evaluation, organized the respective data presentation, and performed the statistical data analysis. ND, MF, and KH-T wrote the first draft of the manuscript. All authors contributed to manuscript revision, read, and approved the submitted version.

## Conflict of Interest Statement

OH and TF were employed by Medovent GmbH, Mainz, Germany. No benefit of any kind will be received either directly or indirectly by the authors. The remaining authors declare that the research was conducted in the absence of any commercial or financial relationships that could be construed as a potential conflict of interest.

## Footnotes

1 http://gratio.efil.de/
